# “Public health is global”: examining Indian stakeholders’ perspectives on Global Health education

**DOI:** 10.1186/s12889-020-09357-2

**Published:** 2020-08-18

**Authors:** Shailendra Sawleshwarkar, Sanjay Zodpey, Joel Negin

**Affiliations:** 1 Faculty of Medicine and Health and Marie Bashir Institute for Infectious Diseases and Biosecurity, Westmead Clinical School, University of Sydney, Sydney, NSW 2006 Australia; 2 Sydney School of Public Health, Faculty of Medicine and Health, University of Sydney, Sydney, NSW 2006 Australia; 3 Public Health Foundation of India, New Delhi, India

**Keywords:** Global health, Curriculum, Public health, Graduate education, India

## Abstract

**Background:**

Global health education has attracted significant attention in recent years from academic institutions in developed countries. In India however, a recent analysis found that delivery of global health education is fragmented and called for academic institutions to work towards closing the developing country/developed country dichotomy. Our study explored the understanding of global health in the Indian setting and opportunities for development of a global health education framework in Indian public health institutions.

**Methods:**

The study involved semi-structured interviews with staff of Indian public health institutes and other key stakeholders in global health in India. The interview questions covered participants’ interpretation of global health and their opinion about global health education in India. Thematic analysis was conducted. A theoretical framework developed by Smith and Shiffman to explain political priority for global health initiatives was adapted to guide our analysis to explore development of global health education in Indian public health institutions.

**Results:**

A total of 17 semi-structured interviews were completed which involved 12 faculty members from five public health institutes and five stakeholders from national and multilateral organisations. Global health was viewed as the application of public health in real-world setting and at a broader, deeper and transnational scale. The understanding of global health was informed by participants’ exposure to work experiences and interaction with overseas faculty. Most common view about the relationship between global health and public health was that public health has become more global and both are interconnected. Integration of global health education into public health curriculum was supported but there were concerns given public health was still a new discipline in India. Most participants felt that global health competencies are complementary to public health competencies and build on core public health skills. Employability, faculty exposure to global health and ‘sensitisation’ of all stakeholders were key barriers to offering global health education programs.

**Conclusion:**

Global health as a concept and educational practice is embryonic in India but there is considerable potential to grow in order to ensure that education meets the needs of future practitioners of global health in the context of sustainable development.

## Background

Global health education is attracting considerable attention from academic institutions in developed countries. The last decade has seen exponential growth in the literature about global health [1] and a dramatic increase in the number of educational global health programs at undergraduate and postgraduate levels [1] . The Lancet Commission on “Education of Health Professionals for the 21st Century” highlighted the notion of ‘interdependence’ in the globalised world and stressed the need for a global health workforce that is competent to deal with global health issues [2]. The Commission also presented the need for training courses in global health underlining the fact that many health professionals participate in a common global pool of talent with much greater transnational movement of health professionals. This suggests a need to revisit public health education in this much broader, interdependent context to provide comprehensive understanding of the interdisciplinary field of public health from a global perspective.

A commonly used definition of global health is by Koplan and colleagues who describe “an area for study, research, and practice that places a priority on improving health and achieving equity in health for all people worldwide. Global health emphasizes transnational health issues, determinants, and solutions; involves many disciplines within and beyond the health sciences and promotes interdisciplinary collaboration; and is a synthesis of population-based prevention with individual-level clinical care [3].” Academic institutions are revising the scope of their public health educational programs in the context of global health education. This is with the aim to meet the demand for global health professionals in the context of the Sustainable Development Goals (SDGs) [4]. ‘Global public health’ is viewed by some as ‘new’ public health which requires additional skills and competencies in an interconnected globalised world [5]. One important finding has been the lack of consensus about what constitutes a global health curriculum. This is largely due to variations in definitions of global health in terms of geographical focus, subject matter and normative frameworks [6].

Most global health education programs are in high income countries (HIC) [1] and are offered in various formats from a module or a concentration in global health to a standalone degree program on global or international health [1, 7, 8]. Most of the studies about global health education were conducted in North American and European countries with minimal representation from Asia [6]. Global health centres have been established in low and middle income countries (LMIC) and south–south collaborations have been suggested as a way forward for countries with the greatest burden of disease to have the best opportunity to respond appropriately [9]. It is also advocated that academic institutions reach across geographic, cultural, economic, gender, and linguistic boundaries to develop partnerships for global health education and research [10]. Despite the existence of several courses in public health in the Asia-Pacific region, there is great variation in the global health education curriculum.

The number of Master of Public Health (MPH) programs being offered in India have expanded rapidly in the past two decades. In 2016–7 there were more than 46 MPH programs in India but the curricula of MPH programs is variable and no standard curriculum exists in the country [11]. Global health is in its preliminary stage in India and a recent review by Pati and colleagues has identified global health as a potential new focus area in medical and public health education [12]. This review also examined the curricular landscape of global health education in India and called for a broader public health curriculum that includes global health [12]. This study found that the present delivery of global health education in India is fragmented and called for academic institutions to work towards closing the developing country/developed country dichotomy [12]. The development of a deeper understanding of global health in the Indian setting and the potential opportunities for development of global health education in Indian public health institutions is timely. A discussion on global health education including competencies and curriculum-related issues will provide a way forward for the development of future Indian public health professionals in the context of the SDGs. Our study explored the understanding of global health in the Indian setting and opportunities for the development of a global health education framework in Indian public health institutions.

## Methods

Our study used semi structured interviews to explore global health education in the Indian setting with stakeholders in public health education in India. A total of 17 participants were interviewed between January to May 2018, including clinicians, academics, professionals from the Ministry of Health, professional bodies and large multilateral organisations.

### Participants

The study involved academic members of public health institutes that currently run graduate public health programs such as MPH in India and other key stakeholders in global health in India. We selected potential respondents through purposive sampling to select 17 participants from these two groups. The first group consisted of faculty members of academic public health institutions in India who were either program coordinators of MPH programs or involved in running modules or units on global health in public health courses. Indian public health schools with MPH programs were identified along with those schools that have global health modules or programs in India [12]. We then chose five large schools of public health from different regions of India and aimed to include faculty members ensuring diversity in years of experience in the field. Faculty members belonged to five large public health institutes across India based in Ahmedabad (Western region), Nagpur (Central region), Jaipur (North-western region), Delhi (Northern region) and Manipal (Southern region). Interviews were conducted with faculty members working in public health institutions in Delhi, Ahmedabad, Manipal, Nagpur and Jaipur. Other key stakeholders included senior members from the government sector at a national level and country representatives of large non-governmental and multilateral organisations including the World Health Organisation (WHO) and the Centres for Disease Control (CDC) country offices in India.

Issue of local and foreign gaze is an important consideration in academic global health work [13]. Our research team included senior public health academics who had expertise in public health and global health education in India with a local gaze (SZ) and in international setting with a foreign gaze (JN). The researcher who conducted the interviews (SS) has worked in public health and medicine in Indian and international settings and completed training in qualitative research methods in 2017. With his background in Indian settings and having worked in international settings, interviewing researcher (SS) had a mixed gaze. The interview guide (Additional file 1) was developed based on a literature review and included questions starting with open ended questions and ending with specific questions on global health education and competencies for public health education programs. Pilot interviews were conducted with two faculty members to assess the validity and flow of questions and modifications were made to the interview guide. One of the researchers (SZ) knew a number of participants through their academic network but the interviewing researcher (SS) did not have any relationship with 15 of the seventeen participants and knew two participants prior to the interview. Initial contact with potential participants was made by the researcher via an email which explained the rationale for the study. All the potential participants who were contacted agreed to participate but two participants could not be interviewed due to logistical issues regarding their schedule and travel by the researcher to their workplace. One of the researchers (SS) travelled to India and completed all the interviews face to face after obtaining written consent at the participants' workplace and no other person was present during the interview. The interviewing researcher (SS) had some understanding of the local context due to his background but made conscious efforts not to interpret the statements by the participants and always asked them to clarify their statements. The interviews generally lasted between 25 and 40 min. The interview started with open questions about the interpretation of global health and their opinion about global health education for public health programs. Interview then proceeded to ask about their views on global health education and global health competencies. Participants were then asked to provide their opinion about a set of global health competencies distilled from literature by two of the authors [14] and their relevance to current and future public health professionals in India. The interviews were audio recorded with the written consent of the participants, and the audio recordings were transcribed line by line. In addition, the researcher also made field notes during interview.

### Analysis

We used thematic analysis of qualitative data employing the framework approach described by Ritchie and Lewis [15, 16]. We used the steps in the framework approach as described by Gale et al. [17]. As our study had some specific issues to explore, but we also wanted to explore newer aspects and refine our codes as we analysed the data and hence, we used a coding that combined inductive and deductive approaches. We first developed a set of initial codes based on prior knowledge of the conceptual framework, and then built on this list by coding from the interview and field notes. Interview transcripts and field notes were studied several times to generate an overall impression. Analysis was conducted with data from field notes taken during interviews and transcripts of the audio recorded interviews and codes were generated. For each participant, key sentences were identified and the interpretations of these were then organized into clusters of themes using Microsoft Excel [18]. The coding and analysis were done with five interviews in the first instance and this was discussed with other authors (JN and SZ) to get feedback and agreement on coding framework. Remaining analysis was completed with regular meetings ensuring agreement between researchers.

A theoretical framework developed by Smith and Shiffman [19] to explain political priority for global health initiatives was adapted to guide our analysis to explore development of global health education in Indian public health institutions. The Shiffman and Smith (2007) framework has been applied to global health initiatives, such as maternal and newborn survival [19] and mental health [20]. We believed that this framework would be useful in exploring the current context of global health education and to explore factors that may influence global health education in public health programs in India. We applied the four principal components of Shiffman and Smith’s Framework: 1) actor power; 2) ideas; 3) political contexts (We used ‘educational contexts’ context instead of ‘political’ contexts for this study); and 4) issue characteristics to describe various themes that emerged. These four categories are broken down further into 11 factors which we modified to explain educational context. Original factors are listed below with modifications to the original factors mentioned in the bracket: (1) Policy Community Cohesion (Public Health Education Community Cohesion), (2) Leadership, (3) Guiding institutions, (4) Civil society mobilization (Stakeholder mobilisation), (5) Internal frame, (6) External frame, (7) Policy windows (Curricular context), (8) Global governance structure (Global frameworks), (9) Credible indicators (Important considerations), (10) Severity (Challenges) and (11) Effective interventions. We adapted this framework and associated factors to outline the relevance of various key elements for the development of global health education for graduate public health education in India. We then categorised these themes into eleven factors as described in the framework but modified some of these factors to suit the educational program development paradigm [19]. Description of each of these four components is included in Table 1 and it also includes key themes categorised into the eleven factors.

**Table 1:** Adaptation of Smith and Shiffman’s framework [19] to global health education for graduate public health programs

Dimension	Description	Factors shaping priority
**Ideas**	The ways in which those involved with the issue understand and portray it.	**Internal frame**: • Diversity in views regarding the meaning of global health. • Recognition that public health is now global but concerns regarding need to focus on domestic ‘local’ health. • Global health education essential but diversity on the ways to integrate in the public health curriculum.
		**External frame**: • Sensitization of employers and prospective students required as understanding of global health is poor.
**Actor power**	The strength of the individuals and organizations concerned with global health education.	**Public health education community cohesion**: • No clear global health community exists to drive the agenda.
		**Leadership:** • Orientation and faculty exposure to global health is mixed. • Well-trained faculty with global exposure needed to advance global health curriculum. • International interactions, collaboration and partnerships required to drive global health education.
		**Guiding institutions - Institutional readiness**: • National landscape and priorities: public health education programs are still relatively new in India. • International academic partnerships are manifold and essential to drive global health education.
		**Stakeholder mobilization**: • Multilateral organizations are potentially a key partner but are not actively engaged in education. • Engagement of potential employers who do not currently understand global health.
**Educational contexts**	The environments in which actors operate.	**Curricular context**: • Foundational public health competencies are essential before building global health competencies. • Limited opportunity to include global health in the existing public health curriculum due to competing priorities. • Views on inclusion of global health in the curriculum ranged from a module to a specialization. Executive global health course after public health program is a preferred option.
		**Global frameworks**: • Need for standardization of global health curriculum across different settings.
**Issue characteristics**	Features of the problem.	**Important considerations**: • Public health education is relatively new in India. • Global health education is embryonic and fragmented. • ‘Local context’ is important for adaptation of global health competency frameworks.
		**Challenges:** • Opportunities for faculty to develop international partnerships and student exchange. • Limited job opportunities and employability concerns for global health professionals.
		**Effective Interventions:** • Partnerships between HIC and LMIC may facilitate effective global health education.

### Ethics

Participation in the study was voluntary, and written informed consent was obtained from the participants prior to the interviews. The Ethics Committee of the Indian Institute of Public Health Delhi, Public Health Foundation of India approved the study (Ethics Reference No. IIPHD_IEC_05_2017).

## Results

A total of 17 semi-structured interviews were completed. Twelve interviews involved faculty members from five public health institutes. The remaining 5 interviews involved key stakeholders from national and multilateral organisations. There were 3 females and 14 males among the 17 participants, with an age range of 35 to 70 years. Academic staff members included two lecturers, six associate professors and four professors with experience ranging from 5 years to 45 years in the public health field. All academic staff members had qualifications in public health or related field and half of them were medically trained in addition to public health. During the interviews, several themes emerged representing the varied perspectives of interviewees and these are presented below using adaptation of Smith and Shiffman’s framework to global health education. Table 1 summarises the key findings and outlines factors as they relate to global health education for graduate public health programs in India using an adaptation of Smith and Shiffman’s framework.

### Ideas

The framework theorises ‘ideas’ as “the ways in which those involved with the issue understand and portray it” [19]. It then outlines internal and external frames for development of those ideas – “frames that resonate internally (Internal frame) unify policy communities by providing a common understanding of the definition of, causes of, and solutions to problem. Frames that resonate externally (External frame) move essential individuals and organisations to action, especially the political leaders who control the resources that initiatives need.” We explored internal frame from the perspectives of the faculty of public health institutions. The main factors in the internal frame revolved around the meaning of global health and the global health-public health interface.

#### Diverse views regarding the meaning of global health

There was a diverse range of views regarding the meaning of global health among participants. Global health was viewed as related to globalisation by many participants. Most mentioned that it deals with populations across the world. Non-faculty participants also talked about global health as an intersectoral issue. A typical view was that it is a transnational and intersectoral field, with one participant describing “all health issues which are relevant from both high income and low-income countries - the other dimension I think in global health is that a more multi-sectorial and transdisciplinary approach to address these issues.” (Participant 3).

One participant described global health as an issue across the globe and as a uniting theme “different issues related to health which affect people across the globe comes under global health. But anything which unites us as a group or unites us together you know under a common thread is global health, according to me.” (Participant 1).

While discussing the meaning of global health one participant admitted that “I haven’t thought as much on global health. I have been very concerned about India’s health. India’s health is one-third of global health.” (Participant 12).

#### The global health -public health interface: ‘Public health is global’

**Fig. 1:**
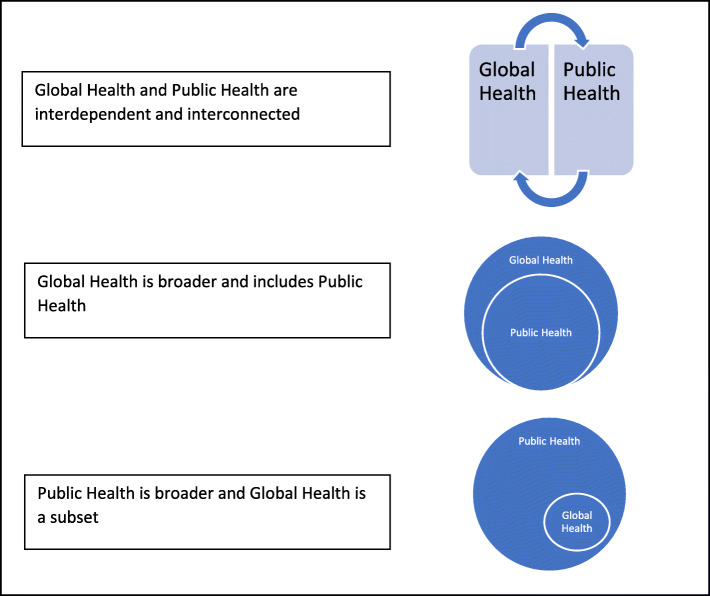
Illustration of participant’s understanding of relationship between global health and public health

The understanding of the relationship between global health and public health was also explored, and again there were differences in participants’ views. Generally, there was agreement that global health was interconnected with public health. Some participants saw global health as the application of public health in a real-world setting at a broader, deeper and transnational scale. The most common view of the relationship was that public health is more global now and both are interconnected, which was shared by 11 participants. Three participants expressed that public health is a subset of global health, whereas another three participants expressed the converse, that global health is a subset of public health. One of the participants mentioned that the complex relationship between public health and global health is best represented as depicted in Fig. 1. (Fig. 1 is influenced by the participant interviews and adapted from a two-dimensional model by Havermann et al. [21]) There was general agreement that public health is essential for global health but also that global health informs public health practice.

One participant described global health as a broader concept “I think public health and global health -- actually global health is more encompassing. So many times, when we talk about public health, I think many of us get restricted in our thinking process, interventions.” (Participant 3).

There was a view by some participants that global health is not different to public health, rather an application of public health at a broader scale. One participant expressed “These are just words, different words. Public health is actually global, you know? If you are learning about public health, then you have to learn global health. So, these are, these go hand in hand. But global health has more to do with the real day-to-day like challenges, so putting those theory into practice is what global health means.” (Participant 1).

#### Sensitisation of external stakeholders

Most of the participants raised the issue of sensitisation of all the stakeholders – faculty, students, employers, national health systems – to global health. A non-faculty expert described global health education this way: “It’s more advocating and dialoguing with the colleges, public health colleges to incorporate that particular agenda and make it more interesting and let them see the value of adding it.” (Participant 9).

Another participant stressed the need to work with all stakeholders so that global health courses prepare students for requirements of the jobs. One non-faculty expert commented “I would say the public health institutes need to work more in sync with the government, more in sync with the agencies that are actually been implementing the programs. Because what happens is once you get out of the course and you join a job, it’s a new world and you get a shock of your life. This is not what I was trained for.” (Participant 14).

### Actor power

Actor power is described as “the strength of individuals and organizations concerned with an issue” and for our study, actors included faculty in schools of public health as well as those working for government and multilateral organisations. These people are important stakeholders for developing the framework for public health and global health education in India. Factors that shape the priority in this component included international experiences of the faculty and faculty exposure to global health programs. Multilateral organisations shape the global health work in Indian setting and they have significant influence on the public health.

#### Orientation to global health – international experiences

The understanding of global health was informed by participants’ backgrounds, education, exposure to work experiences and interaction with overseas faculty. Some of them also related global health to their field of work, research or teaching interest.

Most participants stated that their introduction to global health was provided through international experiences. “I first heard about global health when I started doing my master’s in public health in Glasgow. There was a book which was released at that point of time called ‘Public Health at a Crossroads’ by Robert Beaglehole. I think that was our first introduction to global health.” (Participant 1).

Another participant acknowledged the influence of international partners “Well I first heard global health when I first agreed to co-supervise a student of Harvard University in 2006”. (Participant 4) This was affirmed by a Ministry of Health professional “And you know the context of global health came primarily through the public health emergency related issues - So that initiated this process of global health. And then became the global health security.” (Participant 13).

#### Faculty exposure to global health

Faculty exposure to global health was described as a key constraint to offering global health education programs. One of the most important aspects in developing global health education mentioned by most participants was the need for trained faculty in global health who have exposure to global health issues and who can draw on their own background and experiences.

One faculty member talked about global health exposure and resultant change in approach “How I see global health is probably a shift in thinking or a change of perspective. Thinking doesn’t come naturally to people. You have to be exposed to it in a certain way and there is part of your own education for a start or you have to be working on issues which have a global context.” (Participant 10).

#### Institutional readiness for integration of global health in public health education

Respondents’ had two distinct views on global health education. The first supported the integration of global health education into public health education. The second view suggested that public health is a new discipline in India, and it may be too early to attempt to integrate global health. A respondent from a non-faculty stakeholder shared the view that global health is an important component to be integrated in the public health education curriculum.

Views opposing the inclusion of global health were expressed from academic faculty members. They proposed that public health is a new discipline in the Indian setting and that the curriculum needs to focus on core public health competencies. One of the interviewees mentioned “because the reason is even the public health education itself is quite new in India. And so, you know probably I think it’s too early.” (Participant 15) Another participant was more direct and said “in my view we’re not ready, it’s not the right way to do at this point. Is this the best way to spend our resources?” (Participant 12).

Global health was seen as an essential component in the Indian setting by one faculty member who mentioned “Sometimes when we say about global health, they need to understand the different health systems also. We cannot have our students in isolation. They are -- even in India if you look at, India is a union because health is a state subject and every state is running its own health system. So, it’s better for them to learn from different heath systems across the world” (Participant 11).

#### Role of multilateral organisations

This was particularly important in the context of awareness about multilateral organisations, and large international donor agencies as described by one of the participants “There are a lot of things which we should learn from each other like nowadays it is very important to learn about different multilateral organisations, how they are operating, for example, WHO, for example, UNICEF, United Nations. Even for that matter of fact different philanthropies, they are increasingly becoming a major player, so how they are funding? What is their role in global health? It is very important to be understood because until and unless you have knowledge about these global players, you will not be able to implement things at the local level.” (Participant 1) There was also a view that global health allows more transdisciplinary and intersectoral coordination.

### Educational contexts

We changed ‘political contexts to ‘educational contexts’ and examined factors that may affect educational program development and outlined approaches to global health education as well as competencies that were considered relevant for a global health professional. As described in the original framework regarding policy windows “moments in time when worldwide conditions align favourably for an issue, presenting advocates with especially strong opportunities to reach international and national political leaders”, we explored this dimension in curricular context as well as global frameworks for global health education [19].

#### Approaches to global health curriculum

**Fig. 2:**
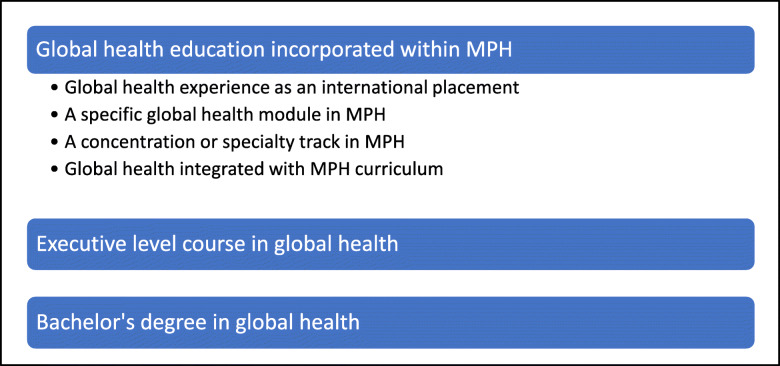
Proposed approaches to global health curriculum offerings in Indian settings

The preferred approach to global health education expressed by participants varied considerably from a module in public health programs to a specialised course on global health as shown in Fig. 2.

Most participants agreed that global health is essential and needs to be introduced in the curriculum for public health education. It was also expressed that if global health is to be offered as a shorter module, the curriculum and training for this is very different from global health delivered as part of a larger program. Participants from multilateral organisations and non-faculty experts suggested the approach of a specialised course on global health for those with a public health background. One expert suggested “MPH - it will be good to have sensitization on global health. A very specific program on global health for people who have already done MPH.” (Participant 2).

There was also a view that there may be some resistance from public health professionals to introduce global health. Public health education in India may be at very early stages and not ready for a new global health dimension, as described by one expert about global health education “But right now we are at a very initial stages, so I don’t think India has evolved itself in this area, it is going to take time.” Yet at the same time others expressed global health may allow clinicians to engage with public health.

Concerns were expressed by some regarding the limited opportunity to include global health in the existing curriculum due to competing priorities. “What should go as a core subject - how many subjects can you fit in and sometimes global health takes a backseat due to competing priorities.” (Participant 1) A faculty member stressed the need for academic institutions to work together to develop global health education “Combining all like-minded institutes willing to incorporate global health within their MPH programs and come together form common competency domain. They can develop learning resource material, tool which is relevant to different context.”

#### Global health education: need for developing core public health competencies

There was recognition that global health requires a set of competencies and most participants felt that these are complementary to public health competencies. One faculty expert discussed global health competencies as being implementation focussed “MPH taught theory but real-time application of these lies in global health. Building on theoretical concepts to apply them in real world.” (Participant 1). Another view was that public health core skills are a prerequisite to becoming a global health professional “see I’d say a person when we say global health, a person first needs to be a public health person. You cannot directly be a global health expert.” (Participant 14).

### Issue characteristics

This component describes particular characteristics of the issue as they relate to priority development. For global health education, the issue of ‘context’ featured prominently along with the issue of employability. Some of the issue characteristics were also covered in the ideas component and there was some overlap in these two components, but we chose to list only those themes that were different in this component. In this dimension, we categorised the themes in important considerations and challenges for implementation of global health education in Indian setting.

#### Context for global health competencies and importance of international partnerships

Most participants agreed with the defined set of competencies distilled from the literature review conducted by the authors in 2017 [14]. Regarding competencies for global health, participants emphasised core public health skills along with skills and knowledge in health system strengthening, global burden of disease and globalisation of healthcare. Some experts stressed the need to develop an understanding of multilateral organisations and big donor agencies as critical for global health education. Adopting a health systems approach, one participant mentioned “to understand about the different health systems will help us to understand where we are lacking.” Participants also felt local ‘context’ is important for global health competencies in public health education.

#### Importance of ‘soft skills’

‘Soft skills’ of political awareness, collaboration and health diplomacy were considered important by some participants, but there was a view that core skills are critical before these soft skills can be considered. One view was that “It is not only the practice, but politics of public health is important. I have learnt biostat, epi, - how do I apply in order to work on a global problem.” Ethics and professionalism were considered to be competencies where context becomes very important.

#### Employability

In addition to faculty exposure to global health, another important challenge expressed by many experts was about job prospects for global health professionals as described by one expert “Important for job prospect- whatever we teach needs to be relevant for jobs. Motivate students that there are jobs in global health. It will take some time. Exposure of faculty to global health - concept, content and issues. Faculty capability is a major hurdle. Second hurdle is job prospect.” (Participant 5) Another expert from the health services sector expressed similar concerns regarding long term employment of the global health courses “there is definitely a need to consider that how this knowledge and skills will be utilised by those who undergo this training. If there is no scope and opportunities, then it won’t sustain.” (Participant 13) Another expert expressed the view that not everyone who takes global health course may get an opportunity to work in global health “But then aspiring that everyone in this global health course to be the person who is leading global health. That’s not going to be happening, that’s reality.” (Participant 12).

## Discussion

This study used interviews from key stakeholders in public health in India to gain valuable insights into global health education in Indian setting. We found that global health is very new as a concept in India and is perceived as a broader version of public health. The number of MPH programs in India has expanded rapidly in the past two decades but there are gaps in public health workforce dynamics in terms of quantity and quality [11, 22]. MPH core competencies have recently been adopted in India (personal communication). Global health education was perceived to be competing with other priorities for curricular space by the public health faculty especially for Indian settings. There was general agreement that a global health component needs to be included in the public health education, but formats varied from a single module to a concentration in global health. There was strong support for an executive style global health course to build on the core public health skills and professional experience.

In our study, most of the participants were exposed to global health due to their involvement in projects funded by multilateral organisations or during their overseas training. Co-supervision of international students was also an area where faculty were exposed to global health. Understanding of global health as an academic discipline and its relationship with public health showed contradictory views. These ranged from global health as part of public health to public health being a subset of global health. The diversity of views on this issue are similar to the back and forth discourse on the meaning of global health, public health and global public health [3, 23–25].

Most interviewees supported the view that global health and public health are interconnected, and global health has a broader transnational context whereas public health has more national focus. This was mentioned in the context of India being a country with 1.3 billion people, and that India has a significant portion of the world’s health problems giving rise to a view that India’s health is global health. This does bring an important dimension of perception of global health by academics and public health practitioners in developing countries; there is a need for global health to accommodate this view. This also illustrates stakeholders’ changing perspectives on global health depending on the context. As discussed by Rowson et al., the position and interests of the actors involved in global health need to be taken into account to develop a global health education framework [26]. There was also acknowledgement that global health informs local health, and, in that context, global health education will be an important consideration for public health education in India. The blurring of boundaries between local health and global health supported the view that “global health is public health” and that the distinction between domestic health and foreign health is dissolving [27]. On the contrary, King and Koski have recently defined global health as “global health is public health somewhere else” and called for more widespread and transparent discussion of the unexamined normative dimensions of global health for practitioners and academic programs [28]. Participants in our study raised the issue of local context with differences between various States in India and hence the need to learn from health systems within the country. There is increasing debate about the issue of ‘transcending geographic boundaries’ as a key component of global health and this discussion is very relevant for India in view of its large population and significant diversity within the country [29].

Discussion about competencies for global health education revealed agreement that global health professionals require a specific set of competencies. Although global health competencies were seen as a distinct entity by most participants, there was general agreement that core public health skills are essential. This view was aligned to the global health competency model described by Ablah et al. which mentions that MPH competencies are considered foundational to the global health competencies, building upon and complementing, rather than replacing them [30]. ‘Soft skills’ (Communication, collaboration, partnering, capacity strengthening and socio-cultural and political awareness) were seen as important but need to be built on core public health skills of program management, analysis and not at the expense of public health competencies. Local ‘context’ was considered important for global health competencies by many stakeholders. This did raise the important question regarding adopting these competencies largely developed by academic institutions in HIC. Eichbaum has discussed the importance of recognising ‘context’ for global health competencies and suggested that these be divided into acquired competencies that can be transferred across contexts and participatory competencies that are linked to contexts [31]. Oni et al. argued for coordination with the academic institutions within Africa and other LMICs for review of global health curricula through a coordinated approach to incorporate local and regional contexts with bilateral exchange for shared learning in a multisectoral context [32]. The opportunity to involve Indian institutions and public health professionals in the discussion about global health curriculum and competencies will make them more relevant to local context. Eichbaum also argued for involving LMIC professionals in the development of global health curriculum with bidirectional flow of ideas and resources [31]. Liu et al. in their review on gaps in global health education pointed to limited research in LMIC and recommended expanding education in global health in developing countries through advocacy and collaboration [6] . Public health academic institutions in LMIC can contribute in application of global concepts to resolve local health issues and integrate context -specific determinants of health [33]. There are already calls that the development of a global health education framework in India should consider building on existing strengths in the country and develop national and global collaborations. This will facilitate sharing of knowledge and expertise on matters related to global health teaching [12]. One of the key constraints identified in our study was limited faculty exposure to global health. National and global collaboration may serve to build local faculty capacity in global health and translate in better student experiences in global health education.

Experts expressed concern regarding jobs for global health students, with lack of awareness among the employers as well as prospective students regarding global health education. This is important with the MPH programs being very new in India and facing challenges in student uptake. A recent review concluded that despite the overwhelming need to strengthen India’s public health workforce, MPH programs are currently undersubscribed [11]. Very few studies have focussed on job opportunities for graduates of global health programs [34, 35]. In view of growth in global health educational programs in HIC, sustainability of global health jobs is being questioned in the context of uncertain demand and supply paradox [36]. The implications of this paradox for global health education programs in LMIC setting is an unanswered question and requires attention.

We also found that stakeholders need sensitisation regarding global health, and this includes prospective students and employers, in order for global health courses to be sustainable. Large numbers of Indian public health professionals are likely to play a more important role in providing health care in domestic and overseas settings in the context of SDGs. India and China are also moving from aid-receiving countries to donor countries. Hence, public health professionals from these major economies will need to develop competencies in global health. An executive style global health program for people with public health educational background and work experience may be an option in settings such as India and may assist them to take the next step in their professional careers.

In our study we included experts from major public health schools, with varied academic and biographic backgrounds. We also included non-faculty stakeholders from government and other multilateral organisations. Participants were key stakeholders in public health education and public health programs in India which allowed for breadth of perspectives. We acknowledge that our sample does not include representations from all the public health schools and includes a small number of non-faculty stakeholders. Our study provides an understanding of global health education in a developing country setting and provides an insight into the opportunities and challenges to strengthen education of global health in public health institutions in this setting but our study did not address global health education in the medical education context. Although our study represents views at this point in time, the landscape of public health education in India may change rapidly in the next few years with more exposure to globalisation and global health. We also acknowledge that we used Smith and Schiffman’s framework that was originally described for political priority for global health issues and that this may have impacted on how we analysed our results.

## Conclusion

Global health as a concept and educational practice is embryonic in India, but there is considerable potential to grow in order to ensure that education meets the needs of future practitioners of global health in the context of sustainable development. It is important to recognise that this study does not aim to define in detail a curriculum development and integration plan for global health education in public health courses in the Indian context. Rather it aims to provide a way forward from which to build appropriate strategies for curriculum transformation for global health education in Indian public health institutions.

## Supplementary information

**Additional file 1.** Interview guide for faculty and other stakeholders.

## Data Availability

The datasets generated and analysed during the current study are not publicly available to guarantee anonymity of the participants. The corresponding author can share further findings and secondary data products on reasonable request.

## Abbreviations

CDC: Centres for Disease Control
HIC: High Income Countries
LMIC: Low and Middle Income Countries
MPH: Master of Public Health
SDGs: Sustainable Development Goals
WHO: World Health Organisation
