# Supplementary material for: “Public health is global”: examining Indian stakeholders’ perspectives on Global Health education
Source: BMC Public Health. 2020 Aug 18;20:1259. doi: 10.1186/s12889-020-09357-2 (PMC7437052; doi:10.1186/s12889-020-09357-2)
Supplement: Supplementary file 1 — Additional file 1. Interview guide for faculty and other stakeholders. [file 12889_2020_9357_MOESM1_ESM.pdf]

Interview guide: Faculty and other stakeholders

Welcome:

Brief overview of the topic and reasons why everyone is here.

Public health professionals remain one of the critical pillars in health workforce for effective performance of health systems. The concept of 'global health' as the health of an interdependent global population is shaping our understanding of which and whose problems we tackle, but also the way in which we educate students, and design the global institutions that govern our collective efforts to protect and promote public health worldwide. This may mean that we need to revisit the public health education in this much broader, globalised interdependent context to provide comprehensive understanding of the interdisciplinary field of public health from a global perspective. Academic institutions are also exploring the scope of their public health educational programs to meet the demand for a global health professional in the context of the sustainable development goals.

Educational competencies are informed by the needs of professional workforce and include a combination of knowledge, skills and attitudes required for acceptable level of practice. Educational competencies are critical to curriculum development and evaluation, coordination across education programs, faculty development and scholarship. Recently, several reports including the Lancet Report on Health Professional education and the WHO report on 'Transforming and Scaling up Health Professionals Education and training' guidelines have highlighted the centrality of competency driven education for bringing in educational reforms.

The aim of this research study is to collect and analyse multi-stakeholder feedback regarding global health education for public health education programs in India.

Introductory questions: Academic and other stakeholders

1. Please introduce yourself briefly.
2. Age:
3. Sex:
4. Educational qualifications:
5. School/University/Organization name:
6. What is your current position within your School/University/Organization?
7. How long have you been working in public health field?
8. How long have you been on faculty/staff at this School/University/ Organization?

Broad questions: Academic and other stakeholders

- When did you first hear the term “Global Health” and in what context?
- How would you define “Global Health”? OR what does global health mean to you?
- What do you think the relationship between Global Health and Public health?

Additional questions for academic faculty:

- Does your School/University offer a master’s level degree in global health (MPH in Global Health/MIPH)
- Does the school/university’s teaching and learning policy/framework/strategy include reference to global health education? Please specify how.
- Does your School/University offer global health content in the masters level public health degree/s?
- And if yes, where and how in the curriculum do students learn about global health in public health courses at your university?
    - A concentration track in public health degree
    - An elective unit/course in public health degree
    - A ‘globalised’ curriculum for the public health degree

Focused questions: Faculty

- What is your opinion about including global health education in the public health curriculum?
- What are the competency domains/themes for global health being taught in the master’s level public health degree at your university?
- What is your opinion about the competency domains listed in this document?
  (Provide global health competency domain table – attached)

  Discussion about each competency domain. Interpretations, relevance, significance for a global health professional.
- Do opportunities/resources currently exist for teaching staff to establish or integrate global health into the current public health curriculum?
- What might be the obstacles/barriers and facilitators to establishing global health curriculum in public health schools/departments?

- How could the curriculum development to enhance global health education be best supported?

Focused questions: Other stakeholders

- What knowledge and understanding of global health does your organisation look for in its public health staff/employees?

- What are the skills for global health your organisation looks for in its public health staff/employees?

- How can the knowledge and skills identified in previous questions help deliver your organization's vision for global health and improve its services?

- How would you suggest global health education at the public health degree programs be enhanced to better address your organization's needs?

- What is your opinion about the competency domains listed in this document? What are the competency domains/themes for global health do you think are relevant in the real-world setting?

  (Provide global health competency domain table - attached)

  Discussion about each competency domain. Interpretations, relevance, significance for a global health professional.

Final closing question: Academic and other stakeholders

- Is there anything else you would like to add on this topic before we conclude the interview?

Thank you for your time today. Please contact me if you have any concerns or questions about the session.

Core competency domains or themes for global health education and summary of the key elements of the competency domains:

Competency Domain	Knowledge (K), Skill (S) and Attitude (A)	Key Elements of the Competency Domain
Domain 1<br>**Global Burden of Disease**	**K**	• Basic understanding of burden of disease in all setting – high middle- and low-income including magnitude, distribution and variations.<br>• Ability to use available data to validate the health status of the population.
Domain 2<br>**Globalization of Health and Health Care**	**K**	• Understanding of different health systems along with understanding of global healthcare trends, human resources for health and role of multiple stakeholders in planning and delivery health services.<br>• Understanding influence of globalisation on health and be cognizant of linkages between local and global health.
Domain 3<br>**Social, Economic and Environmental Determinants of Health**	**K**	• Understand social, economic and environmental factors as determinants of population health.<br>• Key determinants of health and their impact on access to and quality of health services in different contexts and apply it to policy development and problem analysis.
Domain 4<br>**Capacity Strengthening**	**K, S and A**	• Sharing of knowledge, skills and resources to enhance public health programs to build human resource capacity and improve infrastructure.<br>• Strengthen community capabilities, build community partnerships and with community integration improve health of individuals and communities.<br>• Analyze the economic, social, political, and academic conditions and address barriers to produce a strong health workforce.
Domain 5<br>**Ethics and Professionalism**	**K, S and A**	• Understanding of and an ability to resolve common ethical issues and challenges that arise when working within diverse economic, political, and cultural settings to address global health issues.<br>• Evaluation and application of internationals standards and public health ethical frameworks in these settings.<br>• Demonstrate integrity, regard, and respect for others in all aspects of professional practice and optimize the potential of one's scope of practice within the context of a team.
Domain 6<br>**Communication, Collaboration and Partnering**	**S and A**	• Effectively communicate ideas about health and well-being to other professions, community leaders, and the general public.<br>• Communication skills including negotiation, mentoring, conflict resolution, advocacy and liaison.<br>• Multidisciplinary teamwork and team building and working in close collaboration with local institutions

		to design, manage, and evaluate programs in developing countries.
Domain 7 **Health Equity and Social Justice**	**K and S**	• Apply social justice and human rights principles in addressing global health problems.<br>• Demonstrate commitment to global equity, social justice, and sustainable development.
Domain 8 **Program Management**	**K and S**	• Design, implement and evaluate global health program to improve health of individuals and populations in a sustainable manner.<br>• Apply project management techniques throughout program planning, implementation, and evaluation.<br>• Ability to develop and establish relationships and reach collaborative agreements that are mutually beneficial in order to achieve program objectives.
Domain 9 **Sociocultural and Political Awareness**	**S and A**	• Ability to work effectively within diverse cultural settings and across local, regional, national, and international political landscapes.<br>• Being 'Political savvy' – understand historical and present north-south power dynamics and social and political contexts.
Domain 10 **Strategic Analysis**	**S**	• To conduct situational analysis and bring systems thinking and determinants-of-health and population health perspective to analyze a diverse range of complex and interrelated factors to develop context specific intervention to improve global health issues.
Domain 11 **Research Competence**	**S and A**	• Core public health research skills to incorporate qualitative, quantitative, and operations research skills to design and apply reliable, valid, and ethically sound research to identify innovative solutions for global health problems.<br>• Additional specific global health research competencies include identification of actionable determinants, involving communities, partnering with local institutions and respecting cultural diversity.<br>• Translating research to policy and programs.
